# Antibacterial Activity of Fistulin: A Protease Inhibitor Purified from the Leaves of *Cassia fistula*

**DOI:** 10.5402/2012/584073

**Published:** 2012-06-20

**Authors:** I. Arulpandi, R. Sangeetha

**Affiliations:** ^1^Research Department of Microbiology, Asan Memorial College, Chennai 600 100, India; ^2^Department of Biochemistry, School of Life Sciences, Vels University, Chennai 600 117, India

## Abstract

Plant protease inhibitors (PPIs) are one of the important components of a plant's defense machinery. PPIs are active against the insects and microbes which invade the plant. *Cassia* species possess anti-insecticidal and antimicrobial properties and this study was aimed at investigating the antibacterial efficacy of a PPI present in the leaves of *Cassia fistula*. A PPI, fistulin, was isolated from the leaves of *C. fistula* and purified by gel filtration chromatography. The antibacterial activity of the purified fistulin was studied against five bacterial strains, namely, *Bacillus subtilis, Staphylococcus aureus, Klebsiella pneumoniae, Pseudomonas aeruginosa* and *Escherichia coli*. The PPI was found to be very active against *S. aureus, E. coli, B. subtilis,* and *K. pneumonia*, and its efficacy was comparable to the standard drug, streptomycin sulphate.

## 1. Introduction

Proteases are indispensable to the maintenance and survival of plant and animal systems. The proteolytic events catalysed by these enzymes serve as mediators of signal initiation, transmission, and termination in many of the cellular events such as inflammation, apoptosis, blood clotting, and hormone processing pathways [1]. Despite the fact that these enzymes are indispensable to the cells and organisms that host them, they may be potentially damaging when overexpressed or present in higher concentrations. For this reason the activities of these enzymes need to be strictly regulated and controlled [2].

The synthesis of proteases as inactive preproteins and their substrate specificity are factors which monitor their activities. Nevertheless, these do not fulfill the desired level of regulation and therefore cells and organisms require additional means of control. One important control mechanism involves interaction of the active enzymes with molecules that inhibit their activities. These inhibitors partially or completely inhibit their enzymes and are called protease inhibitors (PIs). PIs comprise a large and diverse group of plant proteins capable of forming reversible protein—protein complexes with enzymes resulting in their inactivation [3].

Protease inhibitors are ubiquitous in nature and are found to be involved in various important biological functions like digestion of proteins, control of blood clotting, apoptosis, and signaling receptors interaction in animal. PIs are of common occurrence in the plant kingdom. Plant PIs (PPIs) are generally small proteins or peptides that occur in storage tissues, such as tubers and seeds and also in the aerial parts of plants [4]. PPIs are usually considered to be endogenous proteinases regulators and also function as plant defense agents blocking the insect and microbial proteinases [3]. One of the important defense strategies that are found in plants to combat predators involves PPIs which are, in particular, effective against phytophagous insects and microorganisms. The defensive capabilities of PPIs rely on inhibition of proteases present in insect guts or secreted by microorganisms, causing a reduction in the availability of amino acids necessary for their growth and development [5]. *Cassia* species are traditionally acclaimed for their medicinal values, and many plants belonging to this species possess anti-insecticidal and antimicrobial properties [6]. The leaves of *C. fistula* have been used traditionally to treat skin eruptions, eczema, ulcers, and so forth [7]. The leaf extracts were found to possess a remarkable antioxidant and protective activity against hepatocellular toxicity [8, 9]. The present investigation involves the purification of a protease inhibitor from the leaves of *C. fistula* and the determination of its antibacterial activity. 

## 2. Materials and Methods

### 2.1. Plant Material

The plant materials were collected during December 2009 from Chennai, India. The plant material was duly authenticated by Dr. Jayaraman, Director at National Institute of Herbal Science (PARC), Chennai (Voucher number: PARC/2009/481).

### 2.2. Preparation of Crude Extract

Crude extracts were obtained by homogenizing the leaves using cold 0.2 M sodium phosphate buffer, pH 7.0. Homogenates was centrifuged for 30 min at 16,000× g. Supernatants were harvested and stored at 4°C.

### 2.3. Purification of Protease Inhibitor

All the purification processes were carried out at 4°C. The harvested supernatant was subjected to gradient ammonium sulphate fractionation (40–80%). The precipitates were recovered and resuspended in sodium phosphate buffer and assayed for protease activity. Those exhibiting trypsin inhibitory activities were pooled for further use. The pooled fractions were dialysed extensively using 0.2 M sodium phosphate buffer. The dialysate was further purified on a Sephadex G-100 column. Ten milliliters of the dialysate was loaded onto Sephadex G-100 column which had been preequilibriated with 0.5 M Tris HCl buffer containing 0.5 M sodium chloride and eluted with the same buffer at a flow rate of 30 mL/hour. The eluate was analysed for proteolytic inhibitory activity. 

### 2.4. Trypsin and Chymotrypsin Assay

A portion of the purified inhibitor was preincubated for 10 min at 30°C with 25 *μ*g/mL trypsin or chymotrypsin in 0.2 M sodium phosphate buffer, pH 7.0. To determine the residual activity of the enzymes, 2 mL of 1% casein (w/v) was then added to the preincubated mixture and again incubated for 10 min at 30°C. The reaction was terminated by adding 2 mL of 10% TCA. The reaction mixtures were centrifuged for 10 min at 10,000 g. The supernatants were harvested. To 1.0 mL of supernatant, 5.0 mL of 0.4 M sodium carbonate and 1.0 mL of Folin-Ciocalteu reagent (1 : 3 dilution) were added and the absorbance was read at 660 nm. 

### 2.5. Determination of Antibacterial Activity

Antimicrobial activity of the isolated protease inhibitor was tested against two gram-positive bacteria (**Bacillus subtilis** and **Staphylococcus aureus**) and three gram negative bacteria (*Escherichia coli, Klebsiella pneumoniae,* and* Pseudomonas aeruginosa*). The bacteria were maintained on nutrient broth at 37°C. Fresh overnight cultures of inoculum (0.1 mL) of each culture containing 10^8^ cells, were spread on agar plate. Sterile 6.0 mm diameter blank disc was used to impregnate the partially purified (obtained after dialysis) and completely purified (obtained after gel filtration) proteinase inhibitor. Streptomycin sulphate was used as positive control and dimethyl sulfoxide as negative control. The plates were incubated at 37°C ± 2°C for 24 h. The diameter of zone of inhibition (mean of triplicates ± SD) as indicated by clear area which was devoid of microbial growth was measured.

## 3. Results and Discussion

A novel protease inhibitor named “fistulin” was isolated and purified from the leaves of *Cassia fistula*. The crude extract was obtained from the leaves, and the presence of protease inhibitor was studied by analyzing the inhibition of the activity of trypsin. About 500 mg of protein was present in the crude extract, and the inhibition of proteolytic activity was determined to be 8%. Ammonium sulphate fractionation followed by dialysis was performed as a first purification step of the enzyme. The precipitate thus obtained exhibited maximum inhibitory activity of 41%. Thus, the first purification step using ammonium sulphate fractionation yielded a 5-fold pure inhibitor. 

**Figure 1:**
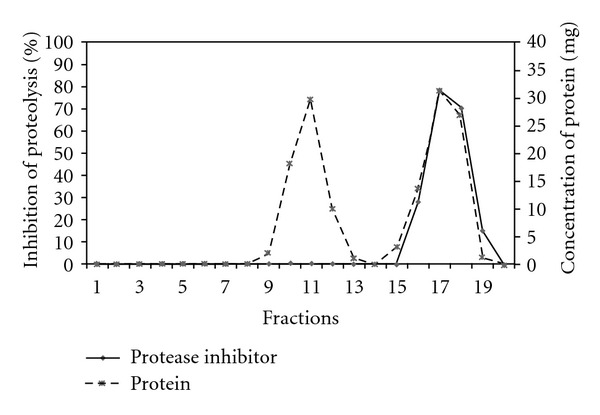
Elution profile of fistulin.

The partially purified inhibitor may contain other proteinaceous contaminants and hence, the inhibitor was subjected to a second step of purification using gel filtration chromatography. The dialysate was loaded on a Sephadex G-100 column and 20 fractions were obtained. Fractions 17 and 18 contained proteins which could inhibit trypsinolysis. Proteins were also present in fractions 10, 11 and 12; however, no inhibitory activity could be observed with these fractions (Figure 1). The fractions 17 and 18 were pooled, and the trypsin inhibitory activity present in the pooled fraction was 74%. This corresponds to 9-fold purification of the inhibitor “fistulin” (Table 1).

**Figure 2:**
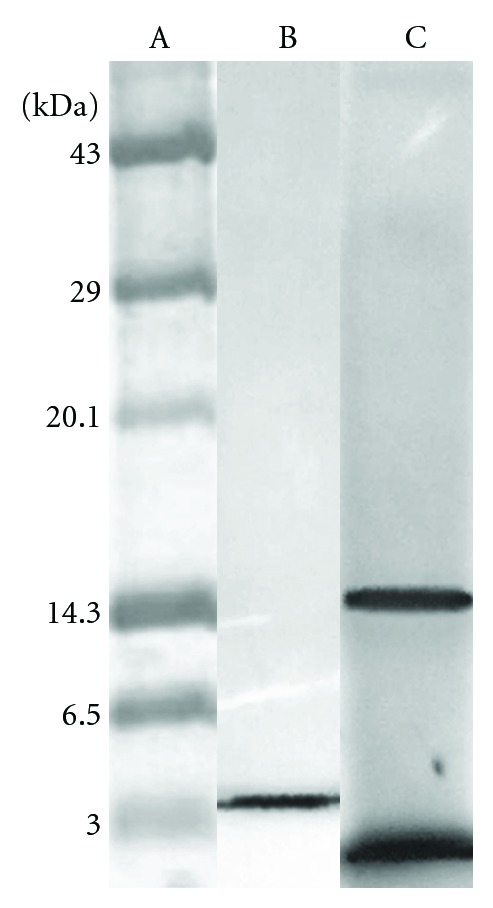
Homogeneity of fistulin. Lane A: molecular weight markers, lane B: purified fistulin obtained after gel filtration chromatography, and lane C: partially purified fistulin obtained after ammonium sulphate fractionation and dialysis.

The homogeneity of the purified inhibitor was studied using sodium dodecyl sulphate polyacrylamide gel electrophoresis. The purified “fistulin” was found to be homogenous by the detection of a single polypeptide. The inhibitor was highly purified as a single band, and this suggested its monomeric nature. The molecular mass of the purified inhibitor was calculated as 4000 Da (Figure 2).

The antibacterial activity of fistulin was evaluated against few pathogenic organisms like *B. subtilis* and *S. aureus, E. coli, K. pneumoniae,* and* P. aeruginosa.* The purified inhibitor was as efficient as the reference standard, streptomycin sulphate (Table 2). The crude extract was less effective on all the tested bacteria when compared to the partially purified and purified fistulin. The purified fistulin showed significant activity against *S. aureus, E. coli, B. subtilis*, and* K. pneumonia.* The highest activity was shown against *S. aureus* and *B. subtilis* and the least activity was against *P*. *aeruginosa *by both the partially purified and purified fistulin. PPIs have a dual role; they suppress the activity of the pathogenic microorganism's protease and also alter its membrane permeability, thus proving to be effective antimicrobial agents [10].

Wijaya et al. [11] have isolated and characterized a trypsin inhibitor from the seeds of *C. fistula *and suggested that the PI may possess anti-insecticidal and antibacterial activities. The antibacterial activity of the bark, seeds, and root of *C. fistula* has been scientifically proved [7]. Ali et al. [12] have isolated three lectins from the seeds of *C. fistula* and the lectins were found to possess antibacterial activity. The antibacterial effect of the leaves of *C. fistula* has been studied by Awal et al. [13]. However, there has been no report on the purification and antibacterial activity of PI isolated from the leaves of *C. fistula.*

## 4. Conclusion

A protease inhibitor named “fistulin” was isolated from the leaves of *C. fistula*. This was a protein purified by ammonium sulphate fractionation and Sephadex G-100 gel filtration chromatography. The inhibitor possessed antibacterial activity and the study was sufficient to confirm the traditional acclaim of the medicinal uses of the leaves of *C. fistula*.

## Figures and Tables

**Table 1:** Purification profile of fistulin.

Purification steps	Extent of inhibition of trypsin (%)	Protein (mg)	Yield (%)	Purification (fold)
Crude extract	8.0	500.2	100	1
Ammonium sulphate fractionation	41.0	161.5	32.3	5
Sephadex G-100 gel filtration chromatography	74.0	77.0	15.4	9.2

**Table 2:** Antibacterial activity of fistulin.

Bacterial species	Zone of inhibition (mm)			
	Crude	Partially purified fistulin	Purified fistulin	Streptomycin sulphate
*Bacillus subtilis*	3 ± 0.12	14 ± 0.30	18 ± 0.4	18 ± 0.43
*Staphylococcus aureus*	4 ± 0.33	13 ± 0.50	18 ± 0.43	18 ± 0.33
*Klebsiella pneumoniae*	3 ± 0.50	10 ± 0.66	14 ± 0.33	14 ± 0.66
*Pseudomonas aeruginosa*	2 ± 1.20	7 ± 0.66	12 ± 0.12	14 ± 0.33
*Escherichia coli*	4 ± 0.33	11 ± 0.43	16 ± 0.66	17 ± 0.26

All the values expressed are Mean ± S.D.
